# Alcohol and the vasculature: a love-hate relationship?

**DOI:** 10.1007/s00424-023-02818-8

**Published:** 2023-05-11

**Authors:** Huige Li, Ning Xia

**Affiliations:** 1 Department of Pharmacology, Johannes Gutenberg University Medical Center, Langenbeckstr. 1, 55131 Mainz, Germany; 2 German Center for Cardiovascular Research (DZHK), Partner Site Rhein-Main, Johannes Gutenberg University Medical Center, Langenbeckstr. 1, 55131 Mainz, Germany

**Keywords:** Alcohol consumption, Red wine, Resveratrol, Disease risk, Cardiovascular disease

## Abstract

Alcohol consumption is a leading risk factor and increases the risk of liver diseases, cancers, tuberculosis, and injuries. The relationship between alcohol use and cardiovascular risk is complex. While it is well established that heavy alcohol use and binge drinking harm cardiovascular health, the effect of light-to-moderate alcohol consumption remains controversial. Observational studies have repeatedly confirmed the U- or J-shaped relationship between alcohol consumption and cardiovascular disease risk, with the lowest risk observed in the light-to-moderate drinking group. However, the protective effect of low-level alcohol has been challenged by recent genetic epidemiological studies with Mendelian randomization. Such studies have their own limitations, and the application of this methodology in studying alcohol has been questioned. Results from the latest Global Burden of Diseases, Injuries, and Risk Factors Study suggest that the impact of alcohol consumption on health depends on the age structure and the distribution of disease burden and underlying causes in a given population. For young adults, even small amounts of alcohol cause heath loss. For older adults facing a high burden of cardiovascular diseases, light-to-moderate alcohol consumption may improve cardiovascular health outcomes. Mechanistically, all types of alcoholic beverages, including wine, spirits, and beer, have been shown to increase the levels of high-density lipoprotein cholesterol and adiponectin, and reduce the level of fibrinogen. Nonalcoholic components of wine, especially polyphenolic compounds like resveratrol, may additionally enhance endothelial nitric oxide production, and provide antioxidant and anti-inflammatory effects.

## Introduction

The relationship between alcohol and health risk is a highly complex and controversial issue. It is well known that alcohol consumption increases the risk of liver diseases, cancers, tuberculosis, and injuries [1, 18]. For the cardiovascular system, however, the situation is complicated. While heavy alcohol consumption and binge drinking are clearly harmful for cardiovascular health, it remains a debate in the scientific literature whether light-to-moderate alcohol drinking lowers the risk of cardiovascular diseases and type 2 diabetes.

## Evidence supporting protective effects of moderate drinking

### Evidence from observational studies

Almost 100 years ago, Raymond Perl reported a U-shaped relationship between the amount of alcohol consumption and mortality. He observed in 1926 a longer lifespan in people drinking moderate amounts of alcohol than both non-drinkers and heavy drinkers [9, 62]. The term “French Paradox” was introduced in 1992 to describe the relatively lower incidence of coronary heart disease in some French populations despite a comparatively high intake of saturated fatty acids [30, 48]. The paradox was attributed to moderate red wine consumption [48].

Later observational studies have repeatedly confirmed the U- or J-shaped relationship between alcohol consumption and cardiovascular disease risk. For instance, a 13-year British study has found the lowest overall mortality in men consuming 8–14 glasses of wine or beer weekly [16]. Small amounts of alcohol are associated with a lower risk of death from ischemic heart disease, and from several other causes [16]. Similarly, a 7-year observation involving 15,400 German participants has led to the finding that men consuming up to 20 g of alcohol per day has the lowest risk of death from cardiovascular causes and death from all causes, with a risk reduction by nearly 50% compared with abstainers [24]. An analysis of 245,207 people in a nationally representative sample of US adults between 1987 and 2000 has found that slightly more than one drink daily is associated with lower cardiovascular mortality. Lifelong abstainers, rare-drinkers, and former drinkers have higher risk of dying from cardiovascular disease than moderate drinkers [36]. A meta-analysis of 84 studies has come to a similar conclusion [50] (Table 1).

**Table 1:** Selected studies on the effects of alcohol consumption

Authors	Study design/no. of Participants	Main outcome
Doll et al. (1994)	Prospective study/12,321	Light-to-moderate alcohol consumption is associated with lower all-cause mortality.
Hoffmeister et al. (1999)	Prospective study/15,400	Light (and possibly moderate) alcohol consumption is associated with lower all-cause and cardiovascular mortality.
Mukamal et al. (2010)	Retrospective study/245,207	Light and moderate alcohol consumptions are inversely associated with cardiovascular mortality.
Ronksley et al. (2011)	Meta-analysis of 84 studies	Light-to-moderate alcohol consumption is associated with a reduced risk of multiple cardiovascular outcomes.
Bell et al. (2017)	Retrospective study/1.93 mio.	Moderate drinking is associated with a lower cardiovascular risk.
GBD (2016) (published 2018)	Meta-analysis/28 mio.	The protective effects of alcohol use associated with ischemic heart disease and diabetes are overweighted by the risks associated with cancers; even small amounts of alcohol use contribute to health loss globally.
Holmes et al. (2014)	Mendelian randomization, meta-analysis of 56 studies/261,991	Rs1229984 A-allele carriage is associated with reduced odds of coronary heart disease in alcohol drinkers but not in non-drinkers.
Millwood et al. (2019)	Mendelian randomization, prospective study/512,715	Conventional analyses show a U-shaped associations of self-reported alcohol intake among men with stroke and myocardial infarction, with the lowest risks at moderate alcohol intake. Genotype-predicted alcohol intake throughout its range is associated with blood pressure, ischemic stroke, and hemorrhagic stroke, without a U-shaped curve.
Biddinger et al. (2022)	Mendelian randomization, cohort study/371,463	Moderate alcohol drinkers exhibit heathier lifestyle factors, adjustments for which reduces cardioprotective associations. Alcohol consumption at all levels is associated with increased risk of cardiovascular disease. Light alcohol consumption is associated with a minimal risk, whereas heavier consumption is associated with an exponentially increased risk of cardiovascular disease.
GBD (2020) (published 2022)	Meta-analysis	Young adults (15–39 years) drinking alcohol have no health benefits, only health risks. For people over age 40, small accounts of alcohol can reduce the risk of cardiovascular disease, stroke, and diabetes.

The above-mentioned results have also been supported by many other studies. Based on such findings, it has been concluded that light and moderate alcohol consumption may reduce the risk of cardiovascular disease. However, the cardioprotective effects of alcohol do not apply to arrhythmia. Alcohol is a risk factor of atrial fibrillation, which is the most common form of arrhythmia [22, 65]. Alcohol consumption increases the rates of paroxysmal and persistent atrial fibrillation.

The effects of alcohol drinking have also been addressed in the Global Burden of Diseases, Injuries, and Risk Factors (GBD) study, which has investigated premature death and disability from over 300 diseases in 195 countries or territories between 1990 and 2016. To generate an improved estimates on deaths and disability-adjusted life-years caused by alcohol, the authors have used 694 alcohol consumption information sources as well as data from 592 prospective and retrospective studies [18]. Also, in this study, the J-shaped relationship has been observed for ischemic heart disease and diabetes mellitus. However, these protective effects are outweighed by the risks of cancers caused by alcohol use. The study concluded that even small amounts of alcohol use contribute to health loss globally; the safest level of drinking is none [18]. Nevertheless, the GBD 2016 study still supports the concept that moderate alcohol consumption protects from ischemic heart disease.

Four years later, results from the GBD 2020 study were published in 2022 [1]. Importantly, the GBD 2020 study applied a novel approach of weighting relative risk according to levels of underlying disease, and took the impact of geography, age, and sex into account. It has found that the threshold level of alcohol on health loss depends on the age structure and the distribution of disease burden and underlying causes in a given population. The health risks associated with low levels of alcohol consumption differ across regions and are larger for younger than for older populations. In young adults with ages of 15 to 39, the risk of cardiovascular disease is low, and injuries are the major cause of health loss. In the population ages 40 to 64, the disease burden shifts to chronic conditions such as cancer. In people older than 65, cardiovascular diseases account for the majority of health loss. The study showed that even small amounts of alcohol are harmful for younger people at age of 15 to 39. For people over age 40, however, health risks caused by alcohol use vary by age and region. Consuming small amounts of alcohol by people in this age group can provide health benefit by reducing the risk of cardiovascular disease, stroke, and diabetes [1]. The authors recommend a modification of existing policy guidelines by recommending alcohol consumption levels by age, no longer by sex [1].

### Evidence from randomized controlled trials

The gold standard to test causal relationships are randomized controlled trials (RCTs), in which confounding factors can be better controlled. However, large-scale RCTs of alcohol use are considered unlikely, or even impossible [20]. Smaller intervention studies indicate that alcohol may have direct effects on the vasculature.

A systematic review and meta-analysis of 63 interventional studies has shown that alcohol increases the levels of high-density lipoprotein (HDL) cholesterol, apolipoprotein A1, and adiponectin. Alcohol also decreases the levels of fibrinogen but does not affect triglyceride levels [6].

In a randomized controlled trail, 224 alcohol-abstaining adults with type 2 diabetes mellitus were randomly assigned to 150 ml of mineral water, white wine, or red wine with dinner for 2 years [19]. Compared with the water group, red wine significantly increased the levels of HDL cholesterol and apolipoprotein A1, whereas white wine decreased fasting plasma glucose level and homeostasis model assessment of insulin-resistance (HOMA-IR) score [19]. Interestingly, only slow ethanol metabolizers (alcohol dehydrogenase alleles [ADH1B*1] carriers) significantly benefited from the effect of both wines on glycemic control, indicating that the effect of wine on glycemic control was mainly driven by alcohol. On the other hand, the stronger effect of red wine than white wine on lipid levels suggests that red wine's effects also involve nonalcoholic constituents [19]. The levels of total phenols (including resveratrol and quercetin) in red wine are known to be 10-fold higher than that in white wine [22].

## Evidence against protective effects of moderate drinking

The relationship between moderate alcohol consumption and health risk has raised a great deal of controversy. The conclusion that moderate drinking protects the cardiovascular system has been challenged by many researchers.

### Lifestyle factors as confounders

Associations in observational studies do not prove causal relationships. An association of moderate alcohol consumption with lower disease risk does not necessarily mean that moderate alcohol drinking reduces disease risk. The study results can be distorted due to confounding [8, 39, 53]. Compared to heavy drinkers and lifetime abstainers, moderate alcohol drinkers are usually well-educated people with healthy lifestyles, higher socioeconomic status, and better physical health [60]. It is supposed that the “health benefits” may be, at least in part, explained by socioeconomic status [60].

Healthier lifestyle behaviors in light-to-moderate drinkers have also been observed in a recent large-scale cohort study [5]. Compared to non-drinkers and heavy drinkers, this group of alcohol consumers shows lower rates of smoking, lower body-mass index, lower red meat consumption, higher physical activity, and higher vegetable intake [5]. Adjustment for these lifestyle factors leads to a reduction of the cardioprotective associations between modest alcohol intake and cardiovascular risk, confirming the contribution of lifestyle factors to the protective effects observed in moderate alcohol drinkers [5]. The authors supposed that adjustments for further confounding factors may even eliminate the residual, cardioprotective associations observed among light/moderate drinkers. This, however, remains debatable. The residual protective effects after adjustment for the known lifestyle factors are still relatively large and statistically significant [5]. Thus, it is rather unlikely that the protective effects of light-to-moderate drinking are completely attributable to lifestyle factors. A recent study has shown that the protective effects of light-to-moderate drinking are independent of demographic measures and health-related behaviors [32]. The association is evident even after further adjustment for a variety of potential explanatory factors, including physical health status, mental health status, socioeconomic status, social support, childhood factors, and earlier life history of alcohol misuse [32].

### The “sick quitter” hypothesis

Another issue is the control group [8]. Current non-drinkers, consisting of never-drinkers and former drinkers, serve as the control group in many studies. It is argued that former drinkers might have stopped drinking because of bad health status or illness. Poorer health, lower socioeconomic status, and less social support have been observed in former drinkers, compared to current drinkers [32]. Thus, the aggregated non-drinking group may be at higher risk, rendering the results artificially to be more favorable for the drinking group. Indeed, a recent large-scale study involving 1.93 million adults has provided evidence for the “sick quitter” hypothesis [4]. Former drinkers have higher risk than never-drinkers in some outcomes including all-cause mortality [4]. Nevertheless, the study results showed that consuming moderate amount of alcohol is associated with a lower cardiovascular risk, even after separation of groups of current non-drinkers, supporting the protective effects of moderate alcohol drinking on the cardiovascular system [4].

### Evidence from Mendelian randomization studies

Recent studies attempt to evaluate cause and effect by using Mendelian randomization by treating genetic background as “quasi-experiments” [20]. Ethanol is converted by alcohol dehydrogenase (ADH) to acetaldehyde, which is detoxified by aldehyde dehydrogenase (ALDH). A genetic variant of the ADH1B gene on chromosome 4 (rs1229984) accelerates the conversion of alcohol to acetaldehyde, causing discomfort after drinking alcohol. Even more relevant is the common loss-of-function variant of the ALDH2 gene on chromosome 12 (rs671). A single copy of rs671 is sufficient to decrease acetaldehyde breakdown enough to produce symptoms [33].

In populations of European descent, only the less important variant (rs1229984) of the two is found and the average carriage rate of rs1229984 A-alleles is about 7% [25]. As expected, carriers of the A-allele of ADH1B rs1229984 consume fewer units of alcohol per week and Rs1229984 A-allele carriage is associated with reduced odds of coronary heart disease [25]. However, this association only exists in alcohol drinkers but not in non-drinkers, indicating that associations (reduced risk) ascribed to the ADH1B variant are mainly due to a reduced alcohol consumption [25]. Further subdivision of the drinker category into light, moderate, and heavy shows the same protective effect of the variant across all alcohol categories. These results lead to the conclusion that reduction of alcohol consumption, even for light-to-moderate drinkers, is beneficial for cardiovascular health [25].

In a prospective study involving 512,715 adults in China with 10 years of follow-up, conventional epidemiological analyses have been compared with genetic epidemiological analyses. The later uses Mendelian randomization to study the effects of the two genetic variants that influence alcohol intake [33]. The conventional analyses have resulted in U-shaped associations between the amount of self-reported alcohol intake with stroke and myocardial infarction in male participants. In consistency with previous observations, moderate alcohol intake is found to be associated with the lowest disease risks. On the other hand, genetic epidemiological analyses have found that genotype-predicted alcohol intake amount throughout its range is strongly associated with blood pressure, ischemic stroke, and hemorrhagic stroke, without a U-shaped curve. These results suggest that moderate alcohol intake provides no substantial protective effects [33].

In a recent cohort study including 371,463 individuals from the UK Biobank, genetic instruments for habitual alcohol consumption have been constructed using the single-nucleotide variants identified in genome-wide association studies. The genetic instrument (restricted alcohol use disorder allele score) is strongly associated with alcohol intake in the UK Biobank [5]. Also, in this study, the previously reported J- or U-shaped association between alcohol intake and cardiovascular risk can be recapitulated. However, the study has found that individuals who consumes light and moderate amount of alcohol have healthier lifestyle behaviors compared with people who do not drink, and has attributed the protective effects largely to healthy lifestyle factors [5]. Moreover, the study results support a nonlinear association between alcohol consumption and cardiovascular risk, with light alcohol intake being associated with a minimally increased risk, and heavier alcohol consumption associated with exponentially increased risks of both hypertension and coronary artery disease [5].

### Limitations of Mendelian randomization studies

Mendelian randomization represents a breakthrough in epidemiological studies. Despite its scientific value, it has also controversies and problems [20]. Mendelian randomization is in fact a simple observational method and has all the risks of any other non-experimental design [37]. Especially, Mendelian randomization methodology is difficult to apply to alcohol studies, as pointed out by researchers [37]. For example, the *ADH1B* rs1229984 variant is associated not only with lower reported alcohol consumption but also with more extensive education [25], thus generating a source of confounding [37]. A second form of confounding can occur if parental genotype is associated with outcome [37], which is the case in alcohol studies. Another issue is the problem of pleiotropy. If a genetic locus can affect outcome through multiple different pathways, then the assumption that genotype is a fair proxy for a single exposure of interest is violated [37]. Both the *ADH1B* rs1229984 variant and the *ALDH2* rs671 variant can lead to acetaldehyde accumulation and greater toxicity after alcohol intake. Thus, the effects of these genetic loci can be reasonably interpreted as effect modification of, rather than merely instruments for, the exposure of interest [37]. Furthermore, by changing alcohol metabolism, *ADH1B* and *ALDH2* polymorphisms influence the exposure to both alcohol and its metabolites. If the metabolites have an impact on the outcome (i.e., the risk of cardiovascular disease), one of the core assumptions underlying mendelian randomization is violated [20]. In short, Mendelian randomization studies have their limitations and the results should be interpreted with cautions. A recent a systematic review of 24 Mendelian randomization studies has found substantial heterogeneity [63], with null associations reported for genetically predicted alcohol consumption with the primary outcomes cardiovascular disease and diabetes [63]. The authors have concluded that it is not yet possible to draw conclusions on the causal role of moderate alcohol consumption on cardiometabolic health. With the continuous advancements in the field of Mendelian randomization, its role in triangulation of evidence may become more and more important [63]. However, Mendelian randomization should not be considered a replacement for a long-term RCT.

## Potential mechanisms

### Vascular function studies in humans

Endothelial function in humans can be assessed non-invasively by the brachial artery flow-mediated dilation (FMD) measurements [13]. Using this technique, it has been shown that heavy alcohol consumption impairs endothelial function with an age-adjusted low %FMD odds ratio of 2.99 [58]. Similarly, binge drinking is associated with impaired vascular function, reducing FMD and enhancing ET-1-induced constriction [21]. Regular moderate-to-heavy intake of alcohol raises blood pressure in humans [35]. On the contrary, the blood pressure falls if moderate-to- heavy drinkers reduce their alcohol intake over periods ranging from 4 weeks to 6 months [47].

In contrast, the effects of low-to-moderate alcohol consumption on FMD are inconsistent. Endothelial function has been reported to be improved [3, 57] or impaired [43, 55] by light-to-moderate alcohol drinking. Other studies have found no changes in FMD [44, 74]. After analyzing 31 published studies, a systematic review article has attributed the inconsistency to multiple reasons including heterogeneity in the pattern and dose of alcohol used as well as subject characteristics [26].

### Animal studies

Alcoholic beverages have been shown to reduce atherosclerosis in rabbit [11, 27], mouse [12, 17, 23], and hamster [64] models. Interestingly, protective effects against atherosclerosis have also been observed for low-dose red wine [40], ethanol alone [12, 17, 64], or dealcoholized red wine extracts [11], indicating that both alcohol and non-alcoholic compounds are effective. Some studies show that red wine is more effective than white wine and whiskey in preventing atherosclerosis, while beer is without effects [27].

### Potential mechanisms

Vasodilation can be caused by alcoholic beverages and by ethanol itself [3]. Human brachial artery diameter increases after one or two alcoholic drinks, and the extent of vasodilation caused by ethanol or wine is comparable [55]. Also, beer consumption is associated with an improvement of vascular function [56].

**Fig. 1:**
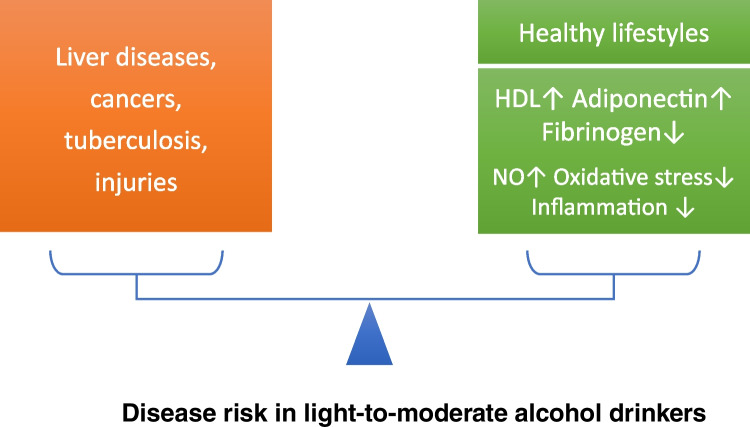
Disease risk in light-to-moderate alcohol drinkers. Alcohol at any level increases the risk of liver diseases, cancers, tuberculosis, and injuries. On the other hand, light-to-moderate alcohol consumption is associated with cardiovascular protective effects, especially in older adults facing a high burden of cardiovascular diseases. Healthy lifestyles observed among light-to-moderate alcohol drinkers contribute to the health benefits. In addition, alcohol at lower levels can provide direct vasoprotective effects, such as elevation of HDL cholesterol and adiponectin, and reduction of fibrinogen levels. These effects have been shown for wine, beer, and spirits, independent of alcohol type. Polyphenolic compounds (including resveratrol) in wine can provide further benefits by stimulating endothelial nitric oxide (NO) production, inhibiting vascular inflammation, and preventing oxidative stress

Moderate dose of alcohol (up to 30 g/day) has been shown to increase adiponectin, HDL cholesterol, and apolipoprotein AI, a main protein in HDL cholesterol [6, 49] (Fig. 1). These effects have been consistently observed in all types of alcoholic beverages [6], including wine, spirits, and beer [6, 56].

Moderate alcohol consumption reduces the circulating levels of fibrinogen [6, 30, 49] (Fig. 1). Red wine and other alcoholic beverages have similar effects so that the beneficial effect on hemostasis is primarily attributable to ethanol [45].

Nevertheless, nonalcoholic components of wine may provide additional benefits, although it is controversial whether the concentration of such compounds is high enough to produce significant effect in vivo [74].

Wine polyphenols are antioxidants and have the potential to prevent low-density lipoprotein (LDL) oxidation, an early step of atherogenesis. Reduced LDL oxidation by wine polyphenols has been shown in human studies [41] and in experimental animals [2, 23]. Moreover, wine polyphenols improve endothelial function and prevent endothelial dysfunction in disease models [42, 52]. Furthermore, polyphenols inhibit angiotensin II-induced upregulation of NADPH oxidase enzymes [51]. At the same time, wine polyphenols increase endothelial NO production [28, 67, 68] (Fig. 1).

Among the wine compounds, resveratrol has received the most attention, both in the scientific community and in the media [54]. Experimental studies have discovered a large range of biological activities of resveratrol in vascular cells [29], including stimulating endothelial NO production [66, 70, 71], inducing mitochondrial biogenesis [10], reducing vascular oxidative stress [29, 61, 72], and inhibiting vascular inflammation [29]. In animal models, resveratrol has been shown to lower blood pressure [46] and suppress atherosclerosis [15, 69].

The results of a number of RCTs with resveratrol are now available. A recent meta-analysis involving 25 RCTs and approximately 600 resveratrol-treated participants has revealed significant effects of resveratrol on glucose and lipid metabolism, decreasing waist circumference, hemoglobin A1c, total cholesterol, LDL cholesterol, and increasing HDL cholesterol [73]. Resveratrol supplementation also improves endothelial function [34]. In patients with cardiovascular disease, resveratrol significantly decreases serum levels of CRP and TNF-α, demonstrating the anti-inflammatory effects of resveratrol [59].

## Conclusion

Alcohol use is a leading risk factor for death and disability. The risk of liver disease, cancers, tuberculosis, and injuries is increased by any amount of alcohol. Alcohol intake also reduces global brain volume measures and regional gray matter volumes [14], and is associated with poor mental health, lack of life satisfaction, and psychological distress [31]. Psychological stress and mental health disorders are themselves also risk factors for cardiovascular disease and may thus enhance the alcohol-induced cardiovascular risk. Indeed, many patients with ischemic heart disease do have multiple risk factors; the interaction of these risk factors may result in risk potentiation. For instance, it has been shown that alcohol consumption and smoking have additive adverse effects on endothelial function and arterial stiffness [7, 38].

The relationship between moderate alcohol consumption and cardiovascular health is complex. Observational studies have repeatedly reported the protective effects of light-to-moderate alcohol drinking on cardiovascular disease risk. Although the conclusion has been challenged by recent Mendelian randomization studies, such studies have limitations and the mothodology needs to be further improved and optimized. Latest results from the Global Burden of Diseases, Injuries, and Risk Factors Study (GBD) 2020 suggest that impact of alcohol consumption on health loss varies significantly across populations and differs strongly by ages. For young adults, even small amounts of alcohol cause heath loss. For older adults facing a high burden of cardiovascular diseases in many world regions, light-to-moderate alcohol consumption may improve cardiovascular health outcomes.

## Data Availability

Not applicable.
